# Database discrepancies in understanding the burden of mass shootings in the United States, 2013–2020

**DOI:** 10.1016/j.lana.2023.100504

**Published:** 2023-05-05

**Authors:** Tristan Bridges, Tara Leigh Tober, Melanie Brazzell

**Affiliations:** Department of Sociology, University of California, Santa Barbara, Santa Barbara, CA, USA

**Keywords:** Mass shootings, Mass killings, Firearms, U.S. gun violence, Gun violence

## Abstract

**Background:**

The United States experiences more mass shootings than any other nation in the world. Various entities have sought to collect data on this phenomenon, but there is no scholarly consensus regarding how best to define mass shootings. As a result, existing datasets include different incidents, limiting our understanding of the impact of mass gun violence in the U.S.

**Methods:**

We compared five datasets of mass shootings for each year included in five databases (2013–2020) and identified overlaps between each database's incidents. These overlaps and divergences between datasets persisted after applying the strictest fatality threshold (four or more) in mass shootings scholarship and policy.

**Findings:**

The datasets collectively include 3155 incidents, but the number of incidents included in each individual dataset varies from 57 to 2955 incidents. Only 25 incidents (0.008% of all incidents) are included in all five datasets. This finding persists even when applying the strictest criteria for mass shootings (four or more fatalities).

**Interpretation:**

Data discrepancies prevent us from understanding the public health impact of mass gun violence. These discrepancies result from a lack of scholarly consensus on how to define mass shootings, likely the downstream consequence of the politicization of gun violence research. We argue for a broad definition of a mass shooting and a government-supported data collection program to remedy these discrepancies. Such steps can improve the quality of research and support policy-making and journalism on the subject.

**Funding:**

This research was supported by the Pahl Initiative on the Study of Critical Social Issues, 10.13039/100007183University of California, Santa Barbara.

**Research in context**

**Evidence before this study**

Mass shootings are an undeniable public health problem in the United States, however, discrepancies between definitions lead to databases with different populations of incidents. Only one peer-reviewed article has compared databases commonly relied upon in research and policy surrounding mass shooting incidents in the United States. That article looks at data discrepancies across four datasets (FBI Supplemental Homicide Report, Everytown for Gun Safety, Gun Violence Archive, Mother Jones magazine) for a single year (2017).

**Added value of this study**

We expand this earlier analysis by including an additional mass shootings dataset (the FBI Active Shooter Report) and widening the scope of analysis to include data from 2013 to 2020, the years for which all five databases have data. This is the first research to undertake a comparative analysis of the data for every year of database overlap available.

**Implications of all the available evidence**

Scholarly, journalist, and policy debates lack a shared definition of mass shootings and, as a result, rely on different databases to make claims. These definitional disagreements hinder an accurate calculation of the public health burden of this form of gun violence in the U.S. Our findings, together with those of the article mentioned above, demonstrate how wide the discrepancies are in which incidents of violence are included in which datasets. The first step in addressing this issue must be to establish a common definition and work toward a common database of incidents. Implementing the definition supported in this article would increase the number of incidents formally counted by researchers, which could help scholars and policy-makers better understand the full burden of these crimes.

## Introduction

In the United States, mass shootings occur more frequently than in any other nation in the world.^1^, ^2^, ^3^, ^4^ As Smart and Schell explain,^5^ however, there is a lack of a scholarly consensus surrounding how to define mass shootings, leading scholars, journalists, policy-makers, and law enforcement agencies to rely on different statistics and figures for understanding mass shootings. Regardless of how they are measured, though mass shootings account for a small fraction of gun violence in every nation, their frequency in the United States makes the nation an international outlier. Yet, the lack of definitional consensus persists at the legal and federal levels. One proximate phenomenon is “mass murder,” which the Federal Bureau of Investigation (FBI) defines as four or more murders occurring without a substantial period of time between murders.^6^ This four fatality threshold has been utilized by many scholars to define mass shootings (i.e., “mass murders” by firearm).^7^, ^8^, ^9^, ^10^, ^11^ In 2013, President Barack Obama called for a separate federal definition of “mass killings” as incidents involving three or more fatalities (with the Investigative Assistance for Violent Crimes Act of 2012) (Of note, the fatality thresholds of both “mass murders” and “mass killings” exclude the possible death of the perpetrator of the violence).

The resulting discrepancies between definitions lead to databases with different populations of incidents, an outcome that is a downstream result of the politicization of gun violence research in the 1990s by the gun rights advocacy group, the National Rifle Association. This organization supported the 1996 Dickey Amendment, which restricted the Centers for Disease Control and Prevention (CDC) from funding gun violence research that promoted gun control. The result is a lack of institutionalized national support for data collection, storage, and accessibility.

These definitional dilemmas shape how researchers operationalize mass shootings in their methodology and analyze these crimes. Yet scholarship has not examined precisely how this dilemma has shaped the body of knowledge on mass shootings.^12^ From a social scientific perspective, this limits what we can learn about these incidents. From a public policy perspective, this allows people to lean on different definitions in politically expedient ways or leaves policymakers confused as to which data are accurate.

The primary aim of this paper is to document database discrepancies between five of the most influential public databases about mass shootings in the U.S. Building on Booty et al.'s^12^ analysis of discrepancies between 2017 data from four databases, we examine five databases for all of the years for which each database includes incidents (2013–2020). Here we provide an analysis of existing datasets that can help adjudicate between both different databases and definitions, helping us better estimate the scale and scope of mass shootings in the United States and make recommendations for best data practices for future research.

## Methods

To perform our analysis, we looked at five of the most widely cited and comprehensive datasets on mass shootings: (1) the FBI's Supplementary Homicide Report, (2) the FBI's Active Shooter Report, and databases amassed by (3) *Mother Jones* magazine, (4) the gun control advocacy non-profit Everytown for Gun Safety, and (5) the non-profit research group Gun Violence Archive. We chose not to include one less commonly cited dataset, Stanford's Mass Shootings in America database, because it had ceased collecting data during the period of analysis. Our methodology builds on the work of Booty et al.,^12^ who compared four of these datasets for the year 2017. We examined a wider period, 2013–2020, expanding on Booty et al.'s analysis significantly. We did not include data from 2021 because federal data collection that year was unusually poor which would have biased our results. States with very large populations that typically submit data to the Uniform Crime Report failed to report data in 2021 in part resulting from challenges related to the COVID-19 pandemic. However, we included an additional database, the FBI Active Shooter Report, which Booty et al. reference in their descriptive statistics but do not include in their analysis of dataset discrepancies. From each of these datasets, we extracted information about the date, state, and city of the shooting, the name of the shooter, and the number of people killed and injured.

(1) The *Supplementary Homicide Report* (SHR)^13^ shares data about U.S. homicides each year. Through the Uniform Crime Reporting Program, the U.S. Federal Bureau of Investigation (FBI) gathers data about murder victim and offender demographics, relationship (if any), as well as information about weapons used and the circumstances of the crime. Noteworthy, the Uniform Crime Reporting website indicates that “justifiable homicides” are not included in SHR data. These account for, but are not limited to, “the killing of a felon by a peace officer in the line of duty” and “the killing of a felon, during the commission of a felony, by a private citizen.”^14^ Data are gathered on a voluntary basis from over 18,000 agencies, including cities, law enforcement, colleges and universities, and tribal agencies. Notably, a voluntary data collection strategy forecloses researchers from assessing the full population of incidents of interest. For our analysis, we subset the SHR data to include only incidents that conform to the FBI's definition of a “mass killing”: a single shooter causing four or more firearm fatalities (excluding the perpetrator), parameters consistent with some common practices in social scientific research on mass shootings.^11^ Technically, the FBI now classifies incidents with three or more fatalities as “mass murders,” but as other scholars have also noted, the four fatality threshold continues to be the most commonly used definition with respect to scholarship on mass shootings and work utilizing SHR data specifically.^5^^,^^12^
(2) *Active Shooter Report (ASR)*.^15^, ^16^, ^17^, ^18^ In addition to voluntary data collection on homicides more broadly, since 2000 the FBI has also gathered data on what it terms an “active shooter”: “an individual actively engaged in killing or attempting to kill people in a populated area.”^15^ In contrast to the FBI's own definition of “mass murder” or “mass killing”, it does not define “active shooter” incidents by any fatality (or injury) threshold. But the reports' discussion of active shooter incidents mentions mass shootings, indicating conceptual overlap.^18^^,^^19^ Active shooter incidents can have multiple shooters but are characterized by randomness, thus excluding domestic, gang, and drug-related violence; self-defence, barricade/hostage, or suicide situations; and shootouts from other crimes.^18^ Data are gathered from internal FBI documents, law enforcement reports, and open-source data, but they acknowledge this does not produce comprehensive coverage, like the SHR, since “there is no mandated database collection or central intake point for reporting active shooter incidents, as exists for other crimes.”^18^
(3) *Mother Jones* (MJ), a progressive U.S. news organization, began building its own publicly accessible mass shootings database and map in 2012.^20^ They used the FBI's fatality threshold (four excluding the shooter) but lowered it to three in 2013 after President Obama mandated a new federal definition for mass killings involving firearms. Because they are specifically interested in “random” or “indiscriminate” shootings by lone perpetrators in public, they exclude “conventionally motivated crimes”^21^ related to robbery, gang violence, or intimate partner violence in domestic settings. This database also excludes crimes where the perpetrator is unknown to law enforcement or media. However, *Mother Jones* does acknowledge that its criteria fails to capture some well-known mass shootings, and even made an exception to include two school shootings with two shooters (at Westside Middle School and Columbine High School).^22^
(4) *Everytown For Gun Safety* (ESG) claims to be the “largest gun violence prevention organization in America,”^23^ formed in 2014 by former New York City mayor Michael Bloomberg to counter the influence of the National Rifle Association on gun policy. The organization's research wing has built a database of mass shootings from 2009 to the present based on media and legal records, including shooters' criminal records.^24^ Everytown's methodology uses the older FBI definition (four or more murder victims, excluding the perpetrator) but does not discriminate by shooting location (public or private), number of shooters, or motive (thus including gang, drug, family and intimate partner, and terrorist violence).
(5) *Gun Violence Archive* (GVA) is a nonprofit research group founded in 2013 that documents in real-time various kinds of gun violence in the U.S., including mass shootings, as well as accidental, self-defensive, and homicides committed by law enforcement.^25^ Like other datasets, GVA includes incidents with a minimum of four victims, not including the perpetrator(s), but includes both injuries and fatalities in this total. The GVA definition is also broader than most, including incidents attributed to gang, family/intimate partner, and drug violence that occur in any location (public or private) or whether incidents occur in multiple locations. GVA gathers data from news sources, including local U.S., national, and international news outlets.

Each dataset draws on different sources and operationalizes “mass shooting” (or the similar concept of “active shooter”) differently, with variation in both the number of perpetrators and victims, as well as locations and motives.

### Analytic approach

After collecting data for the years included in all five datasets (2013–2020), we cleaned and standardized the data for comparison. Initially, we relied on a basic script to identify duplicates across the five datasets for incidents that appeared in each of the five datasets. The script also identified imperfect duplicates where some but not all data overlapped (such as date or location), which we went through individually to assess if they were the same or discrete incidents. Additionally, we subset the data we had gathered by applying the most conservative fatality threshold (four or more) among the datasets’ criteria for inclusion, thus applying a common definition of “mass shooting” across all the datasets. Then we conducted the same analysis for incident discrepancies.

To visualize these overlaps and divergences between the databases, we utilized R to produce three UpSet plots.^21^ UpSet plots allow us to illustrate both data discrepancies between databases and the number of incidents comprising each database.^26^

### Role of the funding source

While this research was funded by the Pahl Initiative on the Study of Critical Social Issues at the University of California, Santa Barbara, they played no role in the study design, data collection, analysis, interpretation, or writing of this manuscript and report.

## Results

### Database descriptive statistics

The number of incidents contained in each of the five databases varies greatly, from Gun Violence Archive with the highest number (2950) and Mother Jones with the lowest number (57). Consistent with Booty et al.,^12^ Table 1 documents these discrepancies in number of incidents in each dataset alongside definitional parameters for inclusion, the total number of fatalities and injuries associated with the incidents included in each dataset.

**Table 1:** Comparing incidents included in five mass shootings datasets, 2013–2020.

Dataset	Criteria for inclusion			Total incidents	Total fatalities	Total injured
	Number of shooters	Number of victims	Characteristics			
FBI SHR	1	4+ killed (excluding shooter)	Subset of overall data using FBI's traditional 4 fatality threshold, any location or motive	210	1071	253
FBI ASR	Any	N/A	Random: No gang, drug, or partner/family violence; no shootouts, self-defense, hostage, or suicide	208	598	1313
Mother Jones	1 (must be known/identified)	3+ killed (excluding shooter)	Random or indiscriminate: No robbery, gang, or partner/family violence	57	449	936
Everytown	Any	4+ killed (excluding shooter)	Any location or motive	157	924	759
GVA	1–2	4+ injured or killed (excluding shooter)	Any location or motive	2950	3166	12,291

**Fig. 1:**
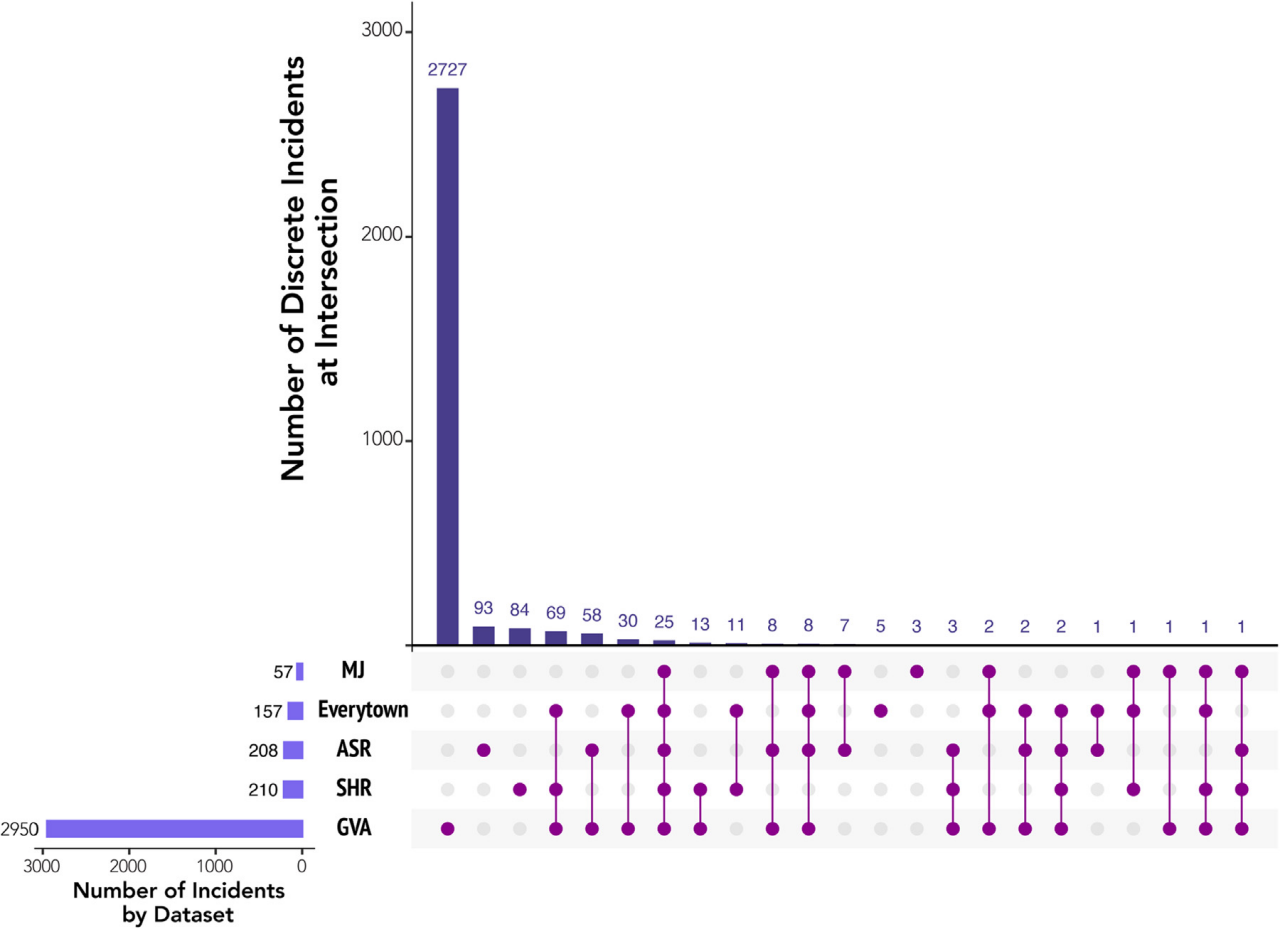
**Incident Overlap between Databases Used to Assess the Burden of Mass Shootings in the U.S., 2013–2020.** Data for this figure were collected from the Federal Bureau of Investigation's “Supplementary Homicide Report” (SHR) and “Active Shooter Report” (ASR), Mother Jones's “Mass Shootings Database” (MJ), Everytown for Gun Violence (Everytown), and Gun Violence Archive (GVA). All databases are publicly available. Calculations and coding of incident overlaps by authors.

Fig. 1 illustrates the frequencies of overlaps between the five different datasets we incorporated into this analysis. Between 2013 and 2020, there were a total of 3155 discrete incidents that were in at least one of the five databases we consulted for this project. Only a total of 25 incidents, however, were included in all five databases (0.008% of all incidents).

**Fig. 2:**
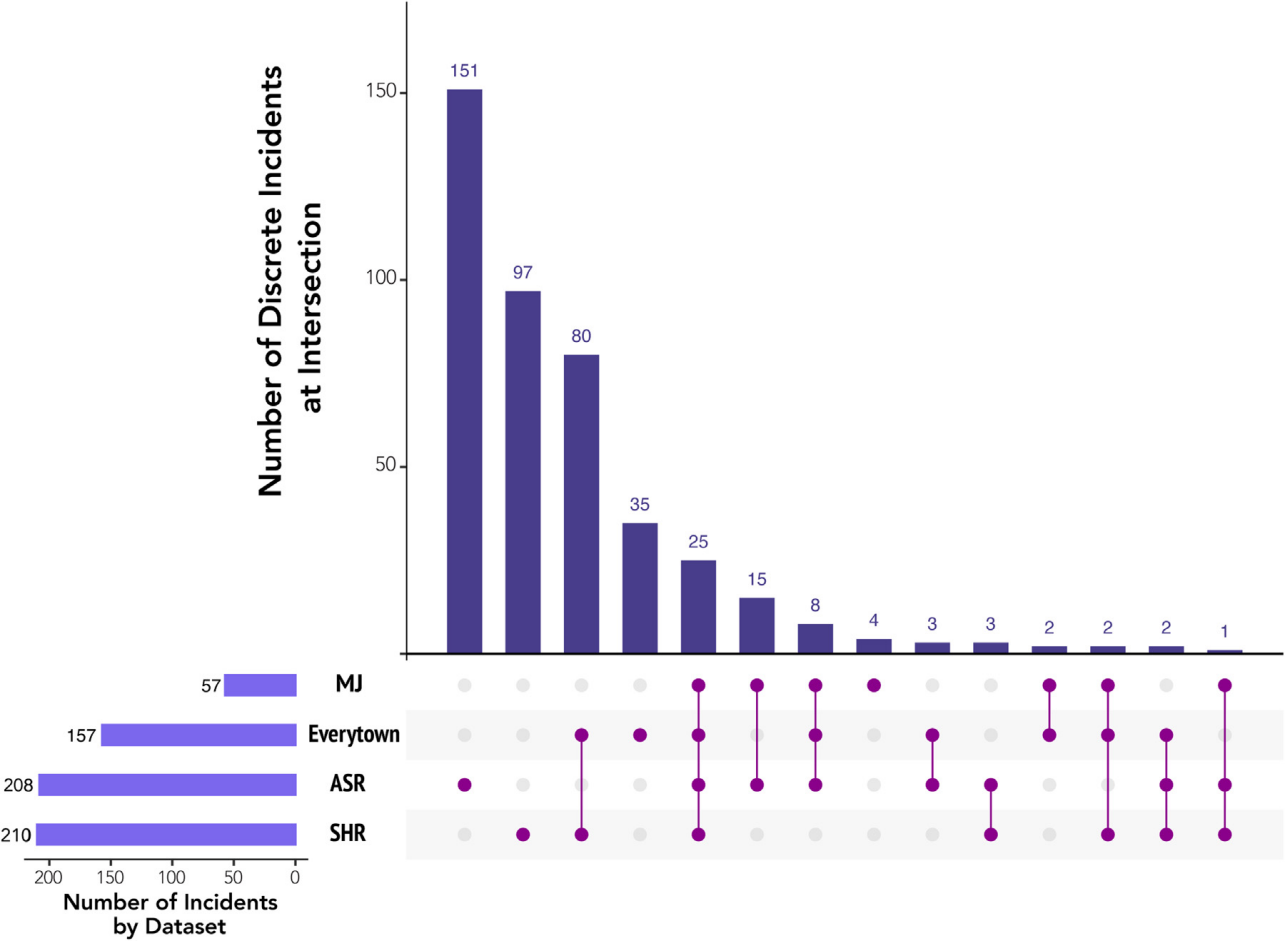
**Incident Overlap between Databases Used to Assess the Burden of Mass Shootings in the U.S., 2013–2020 (excluding Gun Violence Archive).** Data for this figure were collected from the Federal Bureau of Investigation's “Supplementary Homicide Report” (SHR) and “Active Shooter Report” (ASR), Mother Jones's “Mass Shootings Database” (MJ), and Everytown for Gun Violence (Everytown). All databases are publicly available. Calculations and coding of incident overlaps by authors.

These discrepancies could be explained by two datasets with outlier criteria for inclusion. First, the ASR does not have a minimum threshold for victims (injury or fatality). Yet even when excluding the ASR from consideration, the other four datasets (GVA, Everytown, MJ, and SHR) only have a total of 26 overlapping incidents, just one more than when ASR data are included. Secondly, the GVA uses an injury rather than fatality threshold, which accounts for its significantly higher number of incidents in comparison to other datasets. But upon exclusion of GVA data (see Fig. 2), the overlap between the 428 incidents in the four remaining databases remains 25 cases (0.06% of all incidents). While the total population of incidents falls substantially by removing GVA data, substantial discrepancies in incidents persist between databases.

In an additional analysis, we excluded incidents that do not meet the strictest fatality threshold relied upon in existing databases (at least 4 fatalities, excluding the perpetrator). This impacts the number of incidents included in our analysis in some databases more than others. Gun Violence Archive is the most impacted, as their operationalization of mass shootings uses an injury threshold of 4 or more, rather than a fatality threshold.

**Fig. 3:**
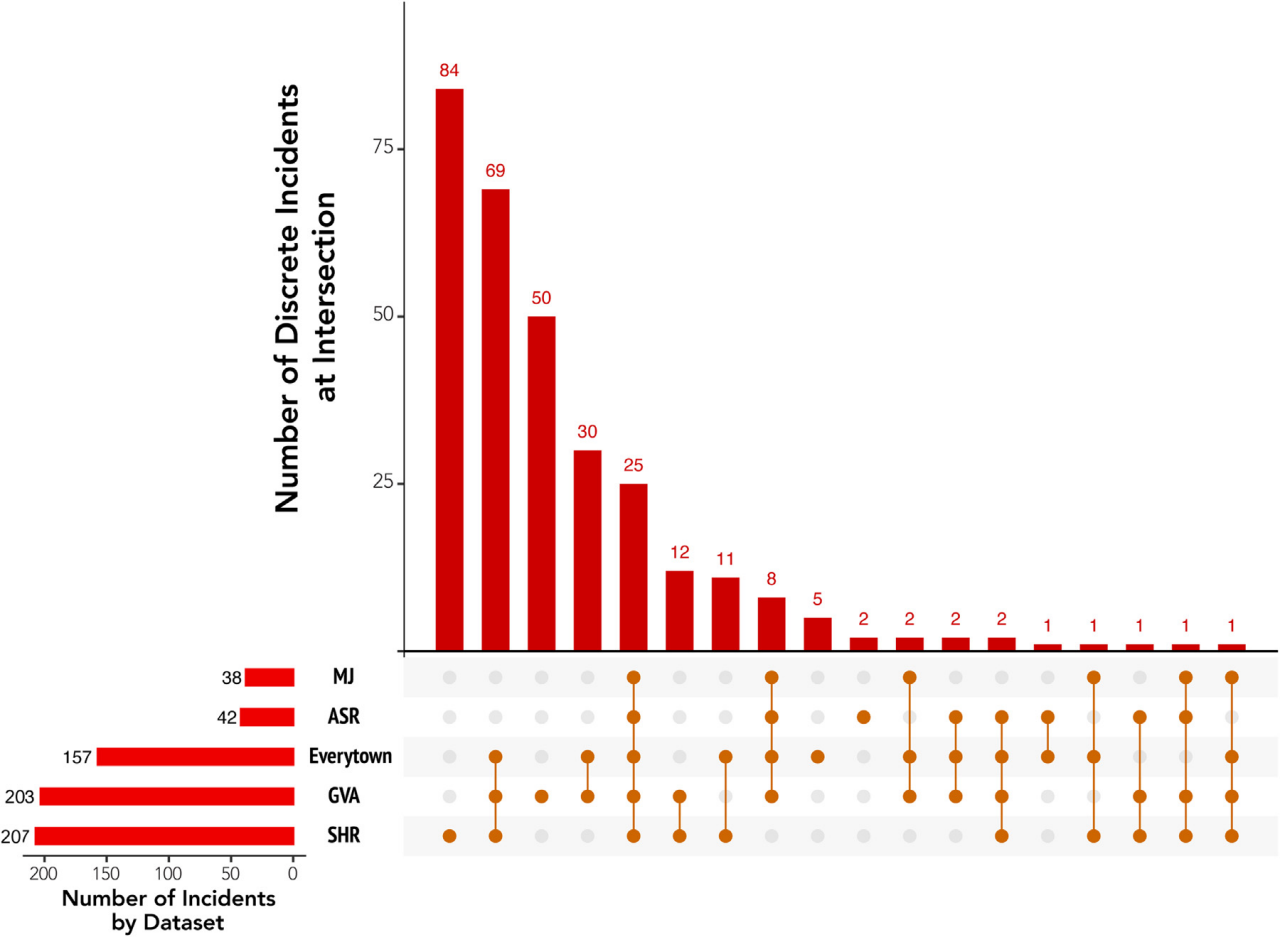
**Incident Overlap with Fatality Threshold of 4+ between Databases Used to Assess the Burden of Mass Shootings in the U.S., 2013–2020.** Data for this figure were collected from the Federal Bureau of Investigation's “Supplementary Homicide Report” (SHR) and “Active Shooter Report” (ASR), Mother Jones's “Mass Shootings Database” (MJ), Everytown for Gun Violence (Everytown), and Gun Violence Archive (GVA). All databases are publicly available. Calculations and coding of incident overlaps by authors.

Table 2 summarizes incidents in each of the five databases included in this analysis that meet the most conservative fatality threshold used in research on mass shootings. Fig. 3 presents data discrepancies among only those incidents meeting the most conservative fatality threshold of at least 4 fatalities. While the total number of incidents is much lower (307), the number of incidents included in all five databases is still the same 25 incidents represented in Figs. 1 and 2.

**Table 2:** Comparing incidents with 4+ fatalities included in five mass shootings datasets, 2013–2020.

Dataset	Total shootings	Total fatalities	Total injured	Excluded incidents in dataset
FBI SHR	210	1067	253	0
FBI ASR	42	406	803	166
Mother Jones	38	392	866	19
Everytown	157	924	759	0
GVA	211	1216	900	2739

The FBI's Supplemental Homicide Report includes non-firearm related murders. In this table, we refer to figures for the subset of the data we built for the purposes of this research based on the FBI's own criteria of “mass murder” (at least 4 fatalities) only including those with one of four separate classifications they include for incidents involving firearms. For more, see Data and Methodology (above).

Thus, conclusions drawn in research can be dramatically shaped by the dataset employed. This problem inhibits our ability to calculate and understand the burden of mass shootings in the U.S.

## Discussion

Expanding on previous work from Booty et al., we examined five databases for all of the years for which each database includes incidents (2013–2020) to understand its discrepancies and overlaps. Our analysis documents a substantial lack of overlap in incidents between databases over a much longer period of time and we incorporate more databases. We document enduring discrepancies across more databases and years than their original analysis. This is problematic because it means that we know less about the burden of mass shootings on U.S. society than a great deal of scholarship suggests.

These findings should not be interpreted as a reliable count of how many mass shootings occurred between 2013 and 2020 in the U.S. Rather, given the varied sources each dataset draws upon and their different operationalization of “mass shooting,” the 3155 incidents identified here offer a less conservative estimate of the number of shootings during this period than any of the databases offers individually. If we are undercounting incidents, these data discrepancies hinder us from knowing by how much. These data show that substantial variation exists in the specific incidents included in five of the most utilized databases for information on mass shootings in the United States. Only a minority of incidents included in any of these databases are included in all of them - even when using the narrowest fatality threshold (4 or more). In our interpretation, this is the result of a lack of scholarly consensus on the definition of a mass shooting, a downstream consequence of the politicization of gun violence research. This has foreclosed thorough and reliable federal data collection, leading organizations to put together their own databases without a centralized form of support and corroboration.

One result of this lack of scholarly consensus is that the category of “mass shooting” is often shaped by assumptions about the perpetrator profile of a mass shooter. The ASR and MJ datasets select data based on a criterion for ‘randomness’ or ‘indiscriminate’ violence, excluding motives related to gang, drug, terrorist, theft, and intimate partner/family violence. In fact, good deal of scholarship on mass shootings excludes incidents associated with gang violence, violence associated with drugs, and family and intimate partner violence as well.^4^ Yet plenty of outlier cases question the validity of this perpetrator profile. For example, the shooters who killed 20 children and seven adults at Sandy Hook Elementary School in 2012 and 18 children and two adults at Robb Elementary School in 2022 both shot family members in domestic settings *before* committing the remainder of their crimes. This is particularly noteworthy as, for instance, Geller et al.'s (2021) analysis of mass shootings incidents from Gun Violence Archive discovered that many incidents are related to family and intimate partner violence which would exclude them from many databases.^4^^,^^27^

The MJ database also excludes crimes where the perpetrator is unknown to law enforcement or the media. This is significant as it excludes unsolved mass shooting incidents, which likely cause underreporting of incidents in low-income areas and areas with larger historically marginalised populations.^28^

When incidents are excluded from the category of mass shootings based on assumptions about perpetrators, data are lost. How we conceptualize who commits mass shootings should not dictate how mass shootings are operationalized; rather, our operationalization of these crimes should dictate our discoveries of who commits them.

Another result of this lack of shared definition is that a majority of what we know about mass shootings is based on analyses of single databases, although our research indicates that no database captures the total population of mass shootings incidents. Additionally, most databases exclude neighbourhood gun violence impacting many individuals and communities which is subsequently not given adequate media and policy attention partially as a result of databases excluding such events. Research on mass shootings rarely includes robustness checks to corroborate findings across datasets, yet our findings indicate that this oversight is critical.

Based on these findings, like Booty et al.,^12^ we advocate for a broader definition of mass shootings as comprising incidents involving one to two shooters with four or more casualties (either *injuries or fatalities*) without restrictions surrounding the number and type of locations or motives. This definition avoids assumptions about shootings or shooters by focusing only on a numerical threshold, basing its criteria on the descriptor “mass.” Narrower criteria have few benefits but important drawbacks, such as severely undercounting incidents. A broad definition does not preclude future scholarship from analyzing subtypes of shootings based on characteristics like motive or strict fatality thresholds. Whether and how incidents differ along these and other types of variation should be answered by scholarship, not conjecture.

We also reinforce past criticisms of media reports as a reliable source of mass shootings data due to media bias,^29^, ^30^, ^31^ and believe a federally coordinated data collection mechanism could provide more legitimate data and facilitate public access. Given the recent specifications of the Dickey Amendment allowing the CDC's new investment in gun violence research, such a mechanism may be on the horizon.

Without a clear definition alongside broad agreement, we are unable to build a knowledge base with the credibility that such a serious and urgent topic requires. Broad agreement on definition and the establishment of a less arbitrarily exclusive definition can only improve the quality of research conducted about mass shootings and an enhanced understanding of the burden of mass shootings for policymakers and legislation.

## Contributors

Conceptualisation: T.B., T.L.T. Data curation: T.B., M.B. Formal analysis: T.B. Funding acquisition: T.B., T.L.T. Methodology: T.B., M.B. Project administration: T.B., M.B. Supervision: T.B. Validation: T.B., T.L.T. Visualization: T.B. Writing (original draft): T.B., T.L.T., M.B. Writing (revising draft): T.B., T.L.T. Review & editing: T.B., T.L.T., M.B. Data for each database were verified by all authors. The initial formal analysis was conducted by T.B. and validated by T.B. and T.L.T.

## Data sharing statement

The data will be fully available to the public without requiring investigator approval and without a data access agreement. All databases used in this analysis are publicly available and can be accessed at any cost.

## Declaration of interests

T.B., T.L.T. and M.B. received financial support for this research from the Pahl Initiative on the Study of Critical Social Issues, University of California, Santa Barbara. There is no conflict of interest associated with this funding.
